# Whole-beam self-focusing in fusion-relevant plasma

**DOI:** 10.1098/rsta.2020.0159

**Published:** 2020-12-07

**Authors:** B. T. Spiers, M. P. Hill, C. Brown, L. Ceurvorst, N. Ratan, A. F. Savin, P. Allan, E. Floyd, J. Fyrth, L. Hobbs, S. James, J. Luis, M. Ramsay, N. Sircombe, J. Skidmore, R. Aboushelbaya, M. W. Mayr, R. Paddock, R. H. W. Wang, P. A. Norreys

**Affiliations:** 1 Department of Physics, University of Oxford, Oxford, UK; 2 Atomic Weapons Establishment, Aldermaston, UK; 3 CELIA, Université de Bordeaux-CNRS-CEA, Talence, France; 4 UKRI-STFC Central Laser Facility, Didcot, UK

**Keywords:** inertial confinement fusion, fast ignition, laser–plasma interactions, plasma channelling, proton radiography, synthetic diagnostics

## Abstract

Fast ignition inertial confinement fusion requires the production of a low-density channel in plasma with density scale-lengths of several hundred microns. The channel assists in the propagation of an ultra-intense laser pulse used to generate fast electrons which form a hot spot on the side of pre-compressed fusion fuel. We present a systematic characterization of an expanding laser-produced plasma using optical interferometry, benchmarked against three-dimensional hydrodynamic simulations. Magnetic fields associated with channel formation are probed using proton radiography, and compared to magnetic field structures generated in full-scale particle-in-cell simulations. We present observations of long-lived, straight channels produced by the Habara–Kodama–Tanaka whole-beam self-focusing mechanism, overcoming a critical barrier on the path to realizing fast ignition.

This article is part of a discussion meeting issue ‘Prospects for high gain inertial fusion energy (part 2)’.

## 1. Introduction

The propagation of intense laser pulses into plasma, and the properties of the plasma structures these pulses leave behind, is an area of active research with a wide range of potential applications.

In underdense plasma, hydrodynamic expansion of an optical field-ionized plasma column forms a low-density channel which has been proposed [1] and demonstrated [2] as a method of guiding high-power driving pulses to obviate the need for capillary guiding structures in high-energy laser Wakefield accelerators. Further, Lemos *et al.* [3] demonstrated production of betatron radiation and inverse Compton scattering from the interaction of picosecond-scale laser pulses with a supersonic gas jet (and in the latter case, a plasma mirror). Future applications of this work will be heavily reliant on the reliable direction of the outgoing radiation. Ensuring repeatability requires channels driven by these picosecond-scale pulses to remain straight throughout the pulse duration. As such, understanding of the channelling processes involved is of paramount importance.

In the near-critical density regime, and particularly in plasma close to a quarter of critical density, channelling by laser pulses is subject to strong hosing and filamentation instabilities which can quickly disrupt the forward progression of the channelling pulse. Mitigation of the hosing instability was investigated by Ceurvorst *et al.* [4] and conditions under which filamentation is suppressed were studied by Matsuoka *et al.* [5]. In particular, relativistic self-focusing effects may mitigate filamentation by focusing a pulse to a spot size smaller than its diffraction limit in a vacuum. If this ‘super-focused’ spot size is comparable to the plasma wavelength, the pulse is resistant to filamentation. This is because it acts similarly to a single filament—its transverse extent is comparable to the spatial scale of plasma density (and therefore refractive index) fluctuations, so where a wider pulse would experience local focusing and defocusing leading to the formation of filaments in regions of focusing, the narrow pulse experiences a more uniform index of refraction across its width and therefore does not filament.

Even if a pulse does survive to propagate beyond the quarter-critical surface, its progress will be slowed once it reaches the critical density surface. The pulse enters the hole-boring regime [6] and its pulse front can only continue to move forward by sustained ponderomotive pressure on the critical density plasma ahead of it. In this regime, continued propagation of the laser is inefficient due to energy reflected from the critical density surface, which acts as a plasma mirror. A sufficiently intense pulse, with dimensionless amplitude/normalized vector potential *a* ≥ 1 will induce a relativistic increase in electron inertia by a factor $\gamma =\sqrt{1+|a{|}^{2}/2}$ (assuming linear polarization) and therefore increase the effective critical density for the pulse by the same factor. This allows such a pulse to propagate further up a density gradient than a lower-intensity pulse of the same wavelength before reaching relativistically critical plasma and entering the hole-boring regime. This effect is known as relativistically induced transparency.

Radiation pressure acceleration of ions in the hole-boring regime was theoretically explored by Robinson [7] and Robinson *et al.* [8] and has been experimentally demonstrated, for example, by Pogorelsky *et al.* [9]. X-ray synchrotron radiation associated with electron acceleration [10] and the effect on the hole boring process of gamma-ray emission accompanying ion acceleration [11] have also been investigated. In all of these potential applications, the ability to reproducibly generate straight channels is again key. In the hole-boring case, strong refraction away from the critical density surface makes this especially difficult to achieve; pulses whose propagation is slightly oblique to the critical density surface will tend to hose away from the forward direction and be reflected.

A particularly pressing application of plasma channelling—which covers a wide range of densities from vacuum to orders of magnitude above classically critical density—is fast ignition inertial confinement fusion (ICF) using a plasma bore-through scheme. Fast ignition promises higher gain than conventional central hotspot ICF while also ameliorating hydrodynamic instability issues that have thus far prevented facilities such as the National Ignition Facility from achieving their goal of thermonuclear ignition [12]. It achieves this by decoupling the compression and heating stages of an ICF implosion and heating the compressed target under isochoric conditions [13] rather than the isobaric conditions [14] typical of conventional hotspot implosions. Tabak *et al.* [12] show that under this isochoric heating model the stringent requirements for achieving a stable implosion are drastically reduced in terms of both compression beam quality and implosion drive symmetry requirements, and that higher gain and favourable scaling can be achieved for realistic laser energy input, compared to more conventional hotspot approaches.

The isochoric (‘fast’) heating stage is, in typical fast ignition variants, delivered to the fuel capsule in the form of a short (order tens of picoseconds), high-energy (tens of kilojoules) laser pulse. How this laser energy is transmitted into the compressed fuel varies between proposed schemes, but usually involves accelerating beams of fast electrons[12] or ions [15], which are able to transport this energy into the dense fuel and deposit it there.

In the plasma bore-through scheme, a laser pulse is used to form a plasma channel and allow the electrons to be produced as close as possible to the central fuel assembly. Use of a single pulse to both bore the channel and deposit energy has been explored [15], as well as schemes using a longer channelling pulse closely followed by a shorter ignitor pulse [16]. Ceurvorst *et al.* [4] found that in the high-intensity regime, use of a single long pulse to form the channel outperforms a multi-pulse scheme. They attribute this to rising and falling edges of sub-pulses resulting in a smaller proportion of the pulses’ total energy being delivered at relativistic intensity, compared to a single pulse with only one rising and one falling edge.

Work carried out at Osaka University’s Institute for Laser Engineering (ILE) by Tanaka *et al.* [17], Kodama *et al.* [18] and Habara *et al.* [19] suggests combining the two relativistic effects—relativistic self-focusing and relativistically induced transparency—described above to increase the penetration depth of ultra-intense laser pulses into fast ignition-relevant plasmas. These plasmas typically contain density gradients ranging from significantly underdense to relativistically critical. First, relativistic self-focusing is used to reduce the spot size of the laser pulse compared to its vacuum diffraction limit. This suppresses filamentation driven by the stimulated Raman scattering (SRS) instability [5].

For the suppression of filamentation, it is desired that the pulse fulfils the condition

1.1 $$2w≲\lambda_{p},$$

as it propagates through the plasma, where *w* is the 1/e^2^ intensity radius of the beam and *λ*_*p*_ the plasma wavelength. The density at which this condition is fulfilled for experimentally realistic spot sizes and Nd:glass or Ti:Sapphire laser systems is usually smaller than the pulse’s quarter-critical density, at which the growth rate of SRS is maximal; for example, a 10 μm diameter spot fulfils this condition at a density of 1.1 × 10^19^ cm^−3^. However, relativistic self-focusing can be employed to constrain the pulse’s spot size beyond its vacuum focal position. At the time of short pulse irradiation, simulations presented in §2b suggest that the electron density at the plasma’s expansion front is of the order 10^19^ cm^−3^, which approximately fulfils equation (1.1). Relativistic self-focusing (which is above threshold for the pulses used in this experiment at densities >10^17^ cm^−3^) will reduce the spot size of the pulse as it propagates; however, the plasma wavelength is also reduced as electron density increases. Assuming that spot size is reduced at least as fast as the plasma wavelength, the Habara–Kodama–Tanaka (HKT) mechanism will protect the pulse from filamentation and enhance its penetration into classically critical plasma.

An experiment, supported by a campaign of simulations, was carried out at the UK Atomic Weapons Establishment’s Orion laser facility to optimize this HKT channelling mechanism in long density scale-length plasma preformed from a planar target.

The organization of the present paper is as follows: §2 describes the methods used in this study: experimental setup and diagnostics in §2a, simulation methods in §2b and a method for synthesizing interferograms to compare to experimental results in §2c. We then present results and interpretation of the interferometry diagnostics in §3a and proton radiographs in §3b, and draw our conclusions in §4.

## 2. Methods

### (a) Experimental setup

**Figure 1.**
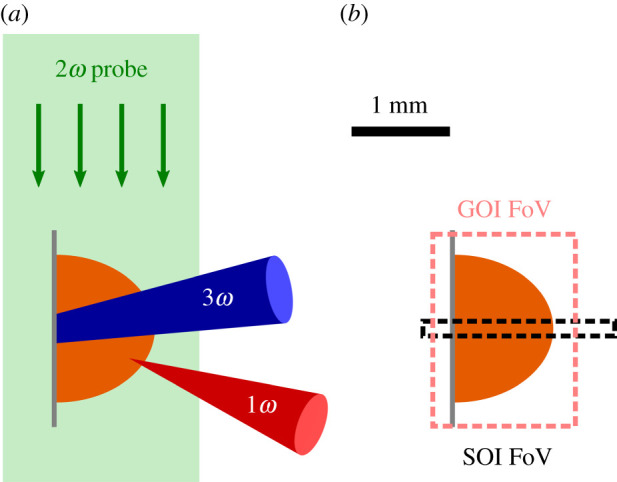
Illustration of the experimental setup used at the ORION laser facility. (*a*) Geometry of the frequency-trebled heating beam, a fundamental frequency short pulse interaction beam and the second-harmonic transverse probe beam. (*b*) Gated and streaked optical camera fields of view. The probe beam is split between the different cameras after passing through an interferometer. (Online version in colour.)

A schematic representation of the experimental setup used at Orion is shown in outline in figure 1. Starting at a time we denote *t*_0_, coronal plasma was ablated from the surface of a 50 μm thick Mylar foil target using a 200 ps ORION long-pulse beam carrying 90 J of energy at the third harmonic for Nd:glass, 351 nm. The beam was incident at 46° from target normal. Interferometric measurements analysed in §3a were consistent with the density profiles found in three-dimensional MHD simulations (detailed in §2bi). These simulations suggest that the laser-produced plasma forms a density scale length of ∼220 μm at times between 550 and 750 ps after *t*_0_, where we define the scale length as

2.1 $$L_{n}=n_{e}|\nabla n_{e}{|}^{-1}=|\nabla \log n_{e}{|}^{-1}.$$

A short pulse was injected into the long scale-length plasma, focused at a position *L* = 800 μm before the original target surface and timed to arrive between 50 ps and 1 ns after the start of long pulse irradiation. Most shots used time delays in the range 550 and 750 ps.

A fundamental (1.053 μm wavelength, 1*ω*) short pulse was employed. Most of these pulses had energies of 100 J, though higher energies of 150 J and 500 J were utilized on a small number of shots. Although pulse duration measurements on 100 J and 150 J pulses were unsuccessful, the Orion laser typically delivers pulses of 500 fs duration at this energy with the parameters used for this experiment. The duration of a 500 J pulse was found to be 1.2 ps.

The short pulse was converted to the second harmonic, 526.5 nm, for a small number of shots. No evidence of channelling was present in any of these shots, but as the available second-harmonic energy (approx. 70 J) was significantly less than that used in the fundamental frequency pulses, a like-for-like comparison is not possible. For this reason, the present publication focuses on the shots taken at the fundamental frequency.

The focal position of 800 μm before original target surface was chosen to inject the pulse near to the plasma expansion front: the short pulse power was sufficient to self-focus in even the lowest densities seen in hydrodynamic simulations so the injection of a pulse at best focus at the expansion front was desired to ensure fulfilment of the condition equation (1.1). At 500 ps delay between long and short pulses, for example, figure 3 predicts injection at electron density of 10^19^ cm^−3^, for which a 10 μm spot size fulfils condition (1.1).

Magnetic fields present in the plasma after the short-pulse interaction were imaged using a proton radiography diagnostic, as laser-produced plasma channels are associated with distinctive magnetic field structures. The setup of this diagnostic is described in §2ai and analysis of experimental images is carried out in §3b.

#### (i) Diagnostic techniques

##### Interferometry

A 527 nm, 50 ns low-power probe pulse (2 MW, 0.6 MW cm^−2^) was used to make interferometric measurements of the preformed long scale-length plasma. Beam splitters were used to deliver the interferograms to three gated optical cameras—providing ‘snapshots’ of the preformed plasma at three different time points—and an optical streak camera which produced a time-resolved picture of a central slice of the interaction. Interferometry was intended to provide a measurement of the plasma density, but in this case the plasma’s density gradients (spanning five orders of magnitude in around 1 mm, see figure 3) were sufficiently large that probe light traversing high-density plasma was refracted significantly and lost without reaching the cameras. As no fringes form in regions of the image which probe light does not reach, this precluded quantitative analysis uses common algorithms. Synthetic interferometry techniques were developed to produce images which were compared to the experimental results, as an alternative. The synthetic interferometry techniques are described in §2c.

We note that angular filter refractometry [20], an optical probing diagnostic designed for near-critical plasmas of long scale length, is a strong candidate as a diagnostic for future experimental campaigns investigating the HKT mechanism.

##### Proton radiography

The second Orion short pulse was used to deliver 200 J of first-harmonic energy to a gold foil, producing by the target-normal sheath acceleration [21–23] (TNSA) mechanism a beam of protons with measured energies up to ∼40 MeV.

The gold foil was positioned 2 cm away from the interaction point, giving protons with energies above 2 MeV travel times ranging from ∼235 ps to ∼1 ns. This range of arrival times at the plasma enables proton probing to resolve the temporal evolution of the interaction.

**Figure 2.**
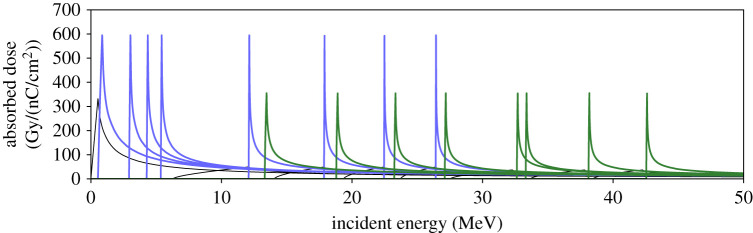
Dose delivered to each active layer of a typical RCF stack (per unit charge fluence) as a function of incident proton energy. Taller, blue peaks are HD810 film and shorter green peaks are EBT3 film. Doses delivered to iron and aluminium filter layers are shown in thinner black lines.Inert substrate and adhesive layers of films are not shown. (Online version in colour.)

A multi-layer stack of radiochromic film (RCF) [24] was used as a detector, placed 20 cm beyond the interaction point. Due to the energy-dependence of ion-solid stopping powers [25], layers of the stack are principally sensitive to different incoming proton energies and therefore can be considered to image the interaction at different times [26,27]. The energy-dependence of a representative RCF stack is shown in figure 2.

As the energy spectrum of TNSA is exponentially decreasing, a much smaller dose is expected to be delivered to rear layers of the stack (which are sensitive to higher energies). To compensate for this, different types of GAFchromic${\;}^{TM}$ RCF were used, with less sensitive films such as HD-810 [28] and HD-V2 [28–30] at the front of the stack and more sensitive films such as EBT3 [28–31] and EBT2 [31,32] at the back. The energy response of an RCF stack used in this experiment is shown in figure 2. Stack energy response can be modified by use of layers of iron as filters between film layers [33]. This increases the incident energy subsequent layers are sensitive to by slowing protons as they pass through the filter layers.

Calibrations of RCF response to dose (M. P. Hill 2016, private communication) were used in a least-squares fitting procedure to estimate the dose distribution delivered to each layer of RCF. The results of this fitting procedure are discussed in §3b.

In principle, it may be possible to use the energy-resolution of the film stack to deconvolve structure present in the proton image on each layer into contributions of specific energies. In practice, however, uncertainties in the dose-extraction procedure as well as inconsistencies between different film types (and even batches of the same film type [31]) make this difficult to implement. We, therefore, attribute all of the structure visible in a proton image to protons in the narrow energy band between the minimum energy able to reach that layer in the stack, *E*_0_ and the minimum energy that can pass into the next layer, *E*_0_ + Δ*E*. This energy range contains protons, whose Bragg peak is located within the layer in question. As long as $E^{-\frac{1}{2}}$, a factor proportional to both the protons’ probing time and the strength of their deflections is near-constant over this interval, image structures will be roughly constant in this energy range.

Having calculated the proton dose delivered to a given layer of film, we can make the approximation that proton fluence through the film at that layer’s ‘Bragg energy’ is proportional to the dose delivered to the film, with proportionality constant uniform across one layer of film. We can then employ a proton radiography inversion program such as PROBLEM [34–36] to determine the deflections experienced by protons which would cause the fluence distribution seen on the film. In this work, we use an implementation of the Monge–Ampère solver proposed by Browne *et al.* [37], a semi-implicit adaptive fixed-point iteration technique. The results of applying this to a layer of RCF are presented in §3b.

### (b) Simulation methods

#### (i) Fluid simulations

Hydrodynamic simulations of the long-pulse interaction were carried out using FLASH 4.6.2 [38–40], a simulation package with support for three-temperature (3T) radiation-hydrodynamics, arbitrary tabulated equations of state and extended magnetohydrodynamics including the Biermann battery mechanism for magnetic field seeding [41]. Simulations were run using the diffusive Local Lax–Friedrichs scheme and ‘crashlike’ 3T energy apportioning behaviour to ensure numerical stability, and magnetic field evolution was handled using the unsplit staggered mesh scheme [42]. A 200 ps laser pulse delivered 90 J of energy to a 300 μm-diameter spot on 50 μm-thick CH foil at a wavelength of 351 nm.

**Figure 3.**
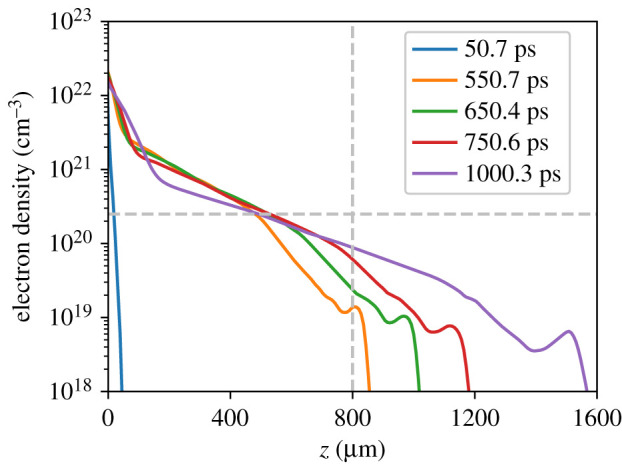
Electron density along the central laser axis in a three-dimensional FLASH simulation at times corresponding to short pulse delivery times. The vacuum focus of the short pulses at 800 μm and quarter-critical density are represented by vertical and horizontal dashed light grey lines, respectively. At the earliest time used, the plasma has a very short scale length of just 6 μm, but at all other drive times the quarter-critical surface is located approximately 500 μm from original target surface. The other times used vary mainly in the progress of the expansion front. Density ‘bumps’ near the end of the density profile are related to the plasma’s expansion into low-density gas, rather than vacuum—a natural limitation of hydrodynamic simulations. (Online version in colour.)

Plasma evolution was tracked for 3 ns following the onset of irradiation. Figure 3 shows an axial line-out of the resulting density profile at times corresponding to the injection of the channelling pulse in different shots. The region of moderately underdense plasma immediately in front of the target has by this time formed a density scale-length of approximately 250 microns. At 1 ns there is a large region of 297 μm scale length between 5 × 10^19^ cm^−3^ and 5 × 10^20^ cm^−3^; at the intermediate times of 550–650 ps an exponential region exists between approximately quarter-critical (2.5 × 10^20^ cm^−3^) and 2 × 10^21^ cm^−3^ density whose scale-length increases from 190 to 220 μm over this period, and a 90 and 110 μm scale-length region between approximately quarter-critical and the expansion front.

**Figure 4.**
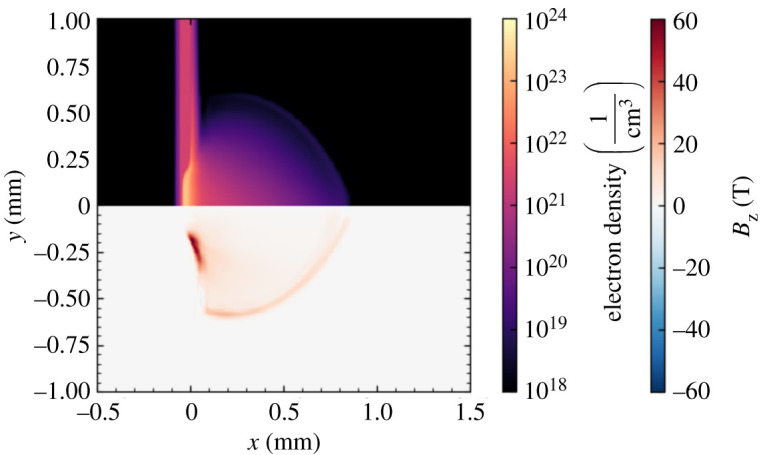
The plasma density profile (upper panel) and magnetic field distribution (lower panel) at *t* = 550 ps, in the *z* = 0 plane of a three-dimensional Cartesian simulation. Magnetic fields are produced at the edges of the laser spot by the Biermann Battery mechanism and then advected out into the expanding plasma. (Online version in colour.)

During long pulse irradiation, misaligned electron density and temperature gradients form in a region around the edges of the laser focal spot, with density varying primarily in the target normal direction and temperature varying more strongly in the transverse direction. In this region, the Biermann battery mechanism [43], a source term added to the MHD equations, generates magnetic fields which encircle the edge of the focal spot. Hydrodynamic motion of the plasma during and after the laser drive advects these magnetic fields out into the expanding plasma, forming a layer of magnetized plasma at the expansion front. After several hundred picoseconds, this field exceeds 10 T in magnitude. An example of the plasma density profile and magnetic field distribution is shown in figure 4.

#### (ii) Particle-in-cell simulations

A campaign of particle-in-cell (PIC) simulations was carried out to support the experiment, using the fully relativistic PIC code Smilei [44]. Firstly, simulations were performed in an ‘azimuthal mode decomposition’ geometry [45] in which a laser matching the parameters of an Orion short pulse was injected into a plasma with electron and ion densities and temperatures extracted from the results of FLASH simulations described in §2bi. The plasma parameters were extracted along a line offset by 20° from the target normal axis, to match the geometry of the experiment. The objective of the simulations was to find a set of parameters (for example, focal position) for which the pulse propagated into the plasma with a minimum of hosing or filamentation (indicating the successful attainment of channelling by whole-beam self-focusing), so that behaviour of parameters that produce favourable results could then be verified in fully three-dimensional simulations. Azimuthal mode decomposition was chosen because it represents an intermediary between cylindrically symmetric and three-dimensional simulation geometries, by expanding the *θ*-dependence of quantities expressed in cylindrical (*r*, *θ*, *z*) coordinates as a truncated Fourier series. The simulations presented here retain the zero-order (cylindrically symmetric) component and the first and second Fourier angular modes of the field.

**Figure 5.**
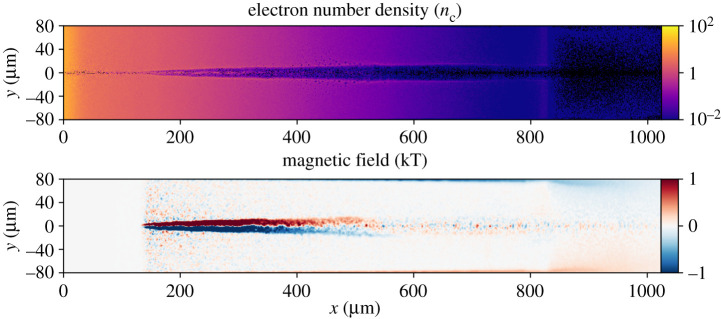
Slice from a cylindrical Smilei simulation 4.4 ps after 200 TW short pulse injection at the right wall. At this time, the pulse energy has been fully absorbed by the plasma. It can be seen that the pulse has produced a low-density channel structure reaching 130 μm from the target surface that expands to approximately 40 μm diameter behind the pulse, but is only around 10 μm wide closer to the target surface (most easily seen from the magnetic field). Original target surface is located at *x* = 0 μm in this simulation. Magnetic fields generated by numerical noise closer to the target surface than the channel reaches have been omitted for clarity. (Online version in colour.)

**Figure 6.**
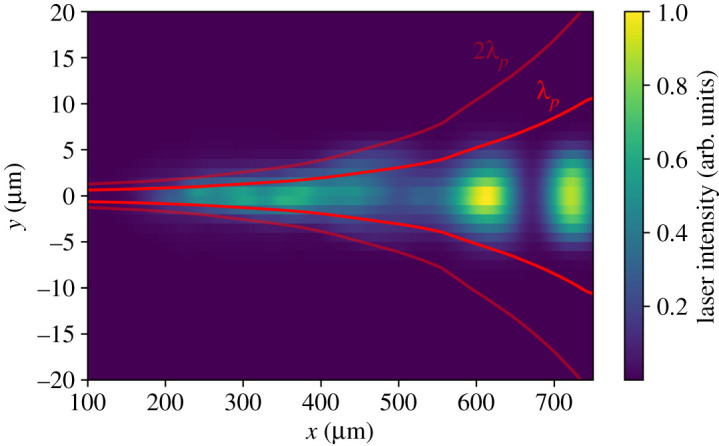
Magnified view of the laser intensity distribution, integrated over the duration of the simulation. It can be seen that, as required by the HKT mechanism, the laser’s energy is mostly confined within a radius of *λ*_*p*_ from the laser axis. (Online version in colour.)

The results of a representative simulation of the short-pulse laser–plasma interaction described above are shown in figure 5. A 100 J, 1 μm laser pulse of duration 500 fs is injected into a plasma with density profile as shown in figure 4. The pulse is initialized at the right wall, *x* = 1024 μm, with a vacuum focal position 800 μm before the original target surface and spot of 5 μm radius. The *x*-axis is defined such that *x* = 0 is the position of the original target surface. These parameters match the vacuum focal position used in the experiment and focal spot size attainable using *f*/3 focusing geometry with 1 μm light. The laser penetrates up to 130 μm before the target surface, as seen in figure 5, and figure 6 demonstrates that most laser energy remains confined within a radius of *λ*_*p*_ from the laser axis as required by the HKT mechanism. A simulation using a more conservative focal spot radius of 15 μm was found to produce qualitatively similar results.

To verify the successful propagation of the laser in these azimuthal simulations, a moving-window three-dimensional Cartesian simulation was run, also using initial plasma conditions extracted from the same 3D FLASH simulation, but with a box size of 200 μm in the longitudinal direction and 80 μm in both transverse directions. Resolution of 8 × 8 × 8 cells per wavelength was used, and while the exact shape of the channel differed slightly from the azimuthal simulations, the qualitative behaviour was comparable. We, therefore, continue with the results of the azimuthal simulations.

**Figure 7.**
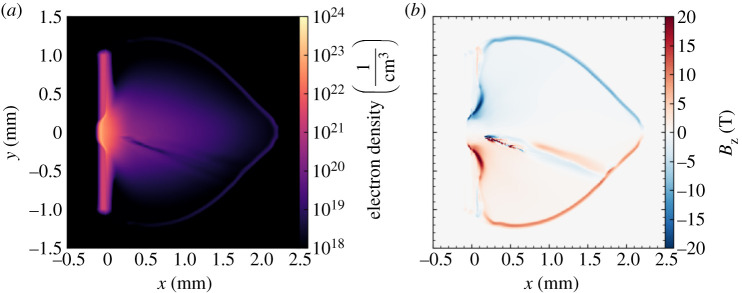
State of the plasma channel structure after 1 ns of hydrodynamic evolution using FLASH, at *t* = 1.55 ns. Slice of three-dimensional MHD simulation at *z* = 0. (Online version in colour.)

Once propagation of the short pulse has finished and all laser energy has been absorbed by the plasma, the channel consists of, to first order, a plasma density structure (reduced density along the short pulse axis with walls of higher density) and an associated azimuthal magnetic field structure. These are shown in figure 5. The density perturbation and magnetic fields associated with a channel are extracted from Smilei outputs and inserted into a FLASH restart file at the short pulse injection time. Electric fields are not included in this transfer of data, as they are not included in MHD models. To ensure validity of this omission, the PIC simulations were allowed to run until significant electric fields associated with the channel had decayed, restoring quasineutrality of the plasma. The magnetic field is smoothed and cleaned of noise to ensure numerical stability of the FLASH simulation once it is restarted. The FLASH simulation is then allowed to continue running, modelling the evolution of channel structures on timescales longer than those accessible to PIC simulations. This allows us to more meaningfully interpret the results of the proton radiography diagnostic, which used probe times between approximately 200 ps and 1 ns after the short pulse propagation. The result of 1 ns of hydrodynamic evolution is shown in figure 7. Most notably the magnetic field strength has reduced significantly, from order kT to order 10 T. Calculation of the magnetic Reynolds number finds results of order unity and the timescale of magnetic diffusion is found to be of order 100 ps, indicating that reduction in field strength is realistic. Some additional non-physical diffusion of magnetic fields may also be present due to use of the Local Lax–Friedrichs scheme. While this process is not analytically rigorous, the assumptions and modelling approaches employed in this analysis are based on reasonable physical motivations, as discussed above, and provide an efficient and reliable way to obtain physically meaningful results.

### (c) Synthetic interferometry calculations

**Figure 8.**
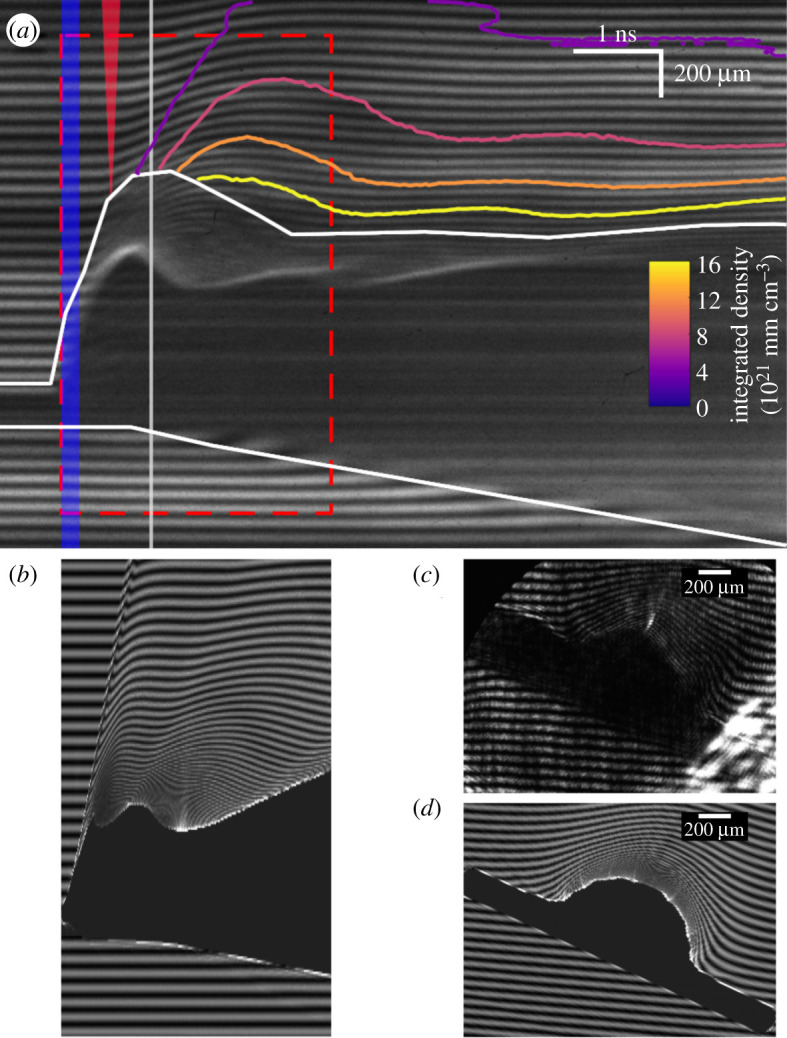
(*a*) Experimental streaked optical interferometry image of the long-pulse interaction, using 10 ns sweep time. The first 1.5 ns recorded, before the interaction begins, are not shown. The region without defined fringes is outlined in white, and recovered density contours are shown outside of this region. These densities agree with the FLASH simulations to within a factor of 3–4. A blue bar indicates the time profile of the heating beam and a red triangle indicates the short pulse time and focal point. (*b*) Synthetic streaked interferometry image produced using the method described in §2c from the plasma density profile extracted from FLASH simulations detailed in §2bi with 526.5 nm probe light. The dashed red box in (*a*) indicates the region corresponding to (*b*). (*c*) Experimental gated interferogram and (*d*) Synthetic gated interferogram taken at *t*_0_ + 1 ns (indicated with white bar in (*a*)). 1 cm of collection lens misalignment is included in (*b*) and (*d*) to optimize agreement with (*a*) and (*c*), respectively. The regions in which light islost are 470 ± 10 μm in the gated images and 600–650 μm in the streaked images. (Online version in colour.)

In order to compare the interferograms acquired by the gated and streaked optical interferometry diagnostics, a synthetic interferometry code was developed. A Fourier-domain analysis algorithm was used to reconstruct the phase shift of the probe beam with results shown in figure 8. While this process was successful in the outer regions of the plasma, refraction by steep density gradients in high-density regions caused probe light passing through these regions to miss the collector lens used to image the interaction at the camera. As no fringes form, it is not possible to recover the density profile in this region. With this in mind, a small python software package was developed in order to produce synthetic interferometry images for comparison to experimental images. This code was written to simulate the effect of refraction as well as fringe formation due to phase shifts.

Firstly, light ray deflections are calculated by performing an integral over the ray propagation direction of the refractive index due to plasma. The integrated refractive index is multiplied by the spatial frequency of the probe laser to yield the phase shift of light passing through this plasma and its gradient in transverse directions is used to calculate the rays’ deflections in the paraxial limit.

In cases where refraction is ignored, the calculated phases are used to determine the interference pattern produced by the probe beam and a fixed reference beam. When refraction is enabled, the ray deflections are also taken into account. When a collection lens is employed, its f-number defines a cone of ray refraction angles within which light remains in the optical system; light refracted at larger angles is lost. The code also simulates relative displacement of refracted rays due to misalignment of the collection lens.

## 3. Results

### (a) Optical interferometry

The data collected by the streaked and gated optical interferometry diagnostics on a typical shot are shown in figure 8.

The streaked interferometry image provides a time-resolved picture of the evolution of the plasma density over 10 nanoseconds, starting prior to the arrival of the heating beams and spatially resolved over 2.25 mm in the target-normal direction.

The dark region does not necessarily correspond to supercritical plasma (for the second-harmonic probe pulse, critical density is 4 × 10^21^ cm^−3^)—as indicated in figure 3 the region above critical density stays confined within 100 μm of the original target surface until at least *t* = 1 ns. Rather, this region indicates large refractive index gradients are present in the plasma, which cause light to refract away from this region and miss the lens which collects probe light. A slightly larger region of probe light loss is present in the experimental interferogram than the simulated image, which may be due to misalignment between the spatial axis of the streak camera and the target-normal axis increasing the apparent feature size in the experimental streak image. Also present in the simulated streaked interferogram is a sharp jump in the fringes just prior to the short-pulse injection time (most visible near the edge of the region without fringes) caused by the density ‘bumps’ at the edge of the expanding bubble; as discussed in figure 3 these bumps are unphysical and accordingly the resulting feature is not present in the experimental interferogram.

The good agreement between experimental and synthetic interferometry images justifies our use of these simulated density profiles in the PIC simulations described in §2bii.

### (b) Proton radiography

**Figure 9.**
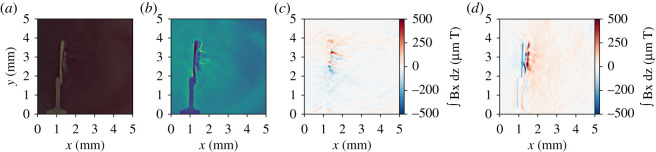
Reconstructed magnetic fields from a shot on which only the long pulses were fired, demonstrating the results of the algorithm in a situation with relatively simple, low-magnitude magnetic field structures. (*a*) A section of raw scanned image from a layer of EBT3 RCF. (*b*) Proton dose (arbitrary units) obtained from (*a*) using dose-response calibrations of (M. P. Hill 2016, Private communication). (*c*) and (*d*) The *x* and *y* components, respectively, of integrated magnetic field density recovered from (*b*) using the algorithm of Brown *et al.* [37]. The integrated field values found here are consistent with the magnitude of long pulse-produced fields near the target surface in figure 7.

The doses received by a stack of RCF were calculated using the procedure described in §2ai. The Monge–Ampère solver of Brown *et al.* [37] was applied to the resulting doses and the result is shown in figure 9. The doses contain large-scale structure due to the intensity modulations of the initial proton beam, which are compensated for using a simple high-pass filtering method. If this pre-processing step is not used, large fictitious transverse magnetic fields manifest, which are attributable to this large-scale beam structure. These large fields can obscure the desired plasma fields, but high-pass filtering used prevents this issue. The more statistically rigorous approach presented by Kasim *et al.* [46] would be worth exploring in future work, for comparison to our simple filter.

**Figure 10.**
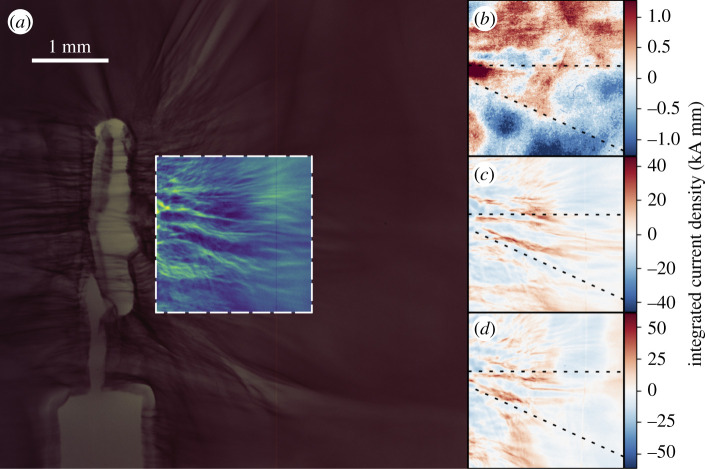
(*a*) A section of an RCF layer. Dose levels (arb. units) are shown within the central region of interest, and the raw scanned image outside. Reconstructed longitudinal integrated MHD current densities are shown in (*b*)–(*d*), for three layers corresponding to energies of (*a*) 1 MeV (1.25 ns), (*b*) 13 MeV (200 ps) (*c*) 23 MeV (105 ps), with times quoted the delay between the arrival of the channelling pulse and the proton probe time. Dashed lines mark the probable location of a channelling feature. Positive integrated current densities correspond to currents counter-propagating with the proton beam, and produce a defocusing effect. (Online version in colour.)

The results of this analysis applied to proton radiographs obtained from channelling shots—in which the short pulse was injected into the plasma—are shown in figure 10. Magnetic fields are observed in a narrow region around the laser propagation axis—a key marker of laser channel formation as demonstrated in figure 5. These fields persist over long times, as figure 7 indicates they are expected to. Panels (*c*) and (*d*) of figure 10 show clear evidence of channel formation matching the opening angle of the *f*/3 beam, and corresponding features are present in panel (*b*), though they are quite washed out. This is likely due to the large relative energy range of protons contributing to the image on the first layer of RCF, from which panel (*b*) is calculated (as indicated in figure 2).

## 4. Conclusion

An experiment was carried out at the UK Atomic Weapons Establishment’s Orion laser with the aim of observing the HKT mechanism of whole-beam self-focusing in fusion-relevant plasma conditions with longer density scale-lengths than have been studied before. Second-harmonic optical interferometric probing was consistent with the plasma conditions found in radiation-hydrodynamic simulations carried out using FLASH, though quantitative interferometric analysis was made difficult by the large densities and density gradients present.

Proton radiography was used to probe the plasma magnetic fields, and quantitative analysis of the resulting images shows evidence of channel formation with a 102 J, fundamental frequency short pulse. The fields associated with the channel persist for times of at least 1 ns as evidenced by the presence of characteristic features in proton radiographs produced by very low-energy protons.

Orion experimental data is © British Crown Owned Copyright 2020/AWE.

## Data accessibility

Third-party codes FLASH 4.6.2 (flash.uchicago.edu/site/flashcode), Smilei 4.4 (maisondelasimulation.fr/smilei) and PROBLEM (github.com/flash-center/PROBLEM) are freely available. Code written for the purposes of this paper and input files for simulations are available at github.com/unpairedbracket/channelling-code. Raw data generated at Orion is only released on an individual basis. Please contact MPH (Matthew.P.Hill@awe.co.uk) to request access.
